# Statin adherence and the risk of Parkinson's disease: A population-based cohort study

**DOI:** 10.1371/journal.pone.0175054

**Published:** 2017-04-07

**Authors:** Violetta Rozani, Nir Giladi, Baruch El-Ad, Tanya Gurevich, Judith Tsamir, Beatriz Hemo, Chava Peretz

**Affiliations:** 1 Department of Epidemiology, School of Public Health, Sackler Faculty of Medicine, Tel Aviv University, Tel Aviv, Israel; 2 Neurological Institute, Tel Aviv Medical Center, Tel Aviv, Israel; 3 Sagol School of Neuroscience, Sackler Faculty of Medicine, Tel Aviv University, Tel Aviv, Israel; 4 Maccabi Healthcare Services, Tel Aviv, Israel; Istituto Di Ricerche Farmacologiche Mario Negri, ITALY

## Abstract

**Background:**

While experimental data provided some compelling evidence on the benefits of statins on dopaminergic neurons, observational studies reported conflicting results regarding the potential of statins to effect the risk of Parkinson’s disease (PD).

**Objectives:**

To evaluate the association between changes in statin adherence over time and PD risk.

**Methods:**

A population-based cohort of new statin users (ages 40-79, years 1999-2012) was derived from a large Israeli healthcare services organization. Data included history of statin purchases and low density lipoprotein cholesterol (LDL-C) levels. Personal statin adherence was measured annually by the proportion of days covered (PDC). PD was detected employing a drug-tracer approach. Stratified (by sex, LDL-C levels at baseline and age) Cox proportional hazards models with time-dependent covariates were used to compute adjusted Hazard Ratio (HR) with 95%CI.

**Results:**

The cohort included 232,877 individuals, 49.3% men. Mean age at first statin purchase was 56.5 (±9.8) years for men and 58.7 (±9.2) years for women. PDC distribution for the whole follow up period differed between men and women: medians 58.3% and 54.1% respectively. During a mean follow up of 7.6 (±3.4) years, 2,550 (1.1%) PD cases were identified. In a 1-year lagged analysis, we found no association between annual statin adherence and PD risk in all age-groups regardless of statin type and potency. Age-pooled HR (95%CI) for men and women with LDL-C levels at baseline ≤160mg/dL were: 0.99 (0.99-1.01), 1.01 (1.00-1.02); and for men and women with LDL-C >160mg/dL levels: 0.99 (0.98-1.01), 0.97 (0.98-1.01).

**Conclusions:**

Our findings suggest that statin adherence over time does not affect PD risk. Future studies should use large-scale cohorts and refining assessments of long-term profiles in statin adherence.

## Introduction

Previous experimental studies demonstrated beneficial biochemical effects of statins on dopaminergic neurons, including suppressed formation of *α*-synuclein aggregation [1] and the development of Lewy-bodies in Parkinson's disease (PD) as well as their anti-oxidant [1] and anti-inflammatory properties [1–3]. Although most observational studies involving large populations (6,465-23,780) found no associations between statin use and PD risk [4–7], findings are inconsistent, varying from significant decrease (43%-63%) [8–13] to significant increase (two fold) [14] in the risk of PD occurrence. This inconsistency could be ascribed to methodological differences in assessing statin exposure such as using self-report questionnaires [8, 9], as a dichotomous use/nonuse variable [9, 10], or by use duration [8, 11, 12]. With a single exception [10], no study considered changes in statin adherence (including changing statin type or dose, and pausing, terminating, or resuming use) occurring during long-term therapy [15]. Similarly, the confounding effect of serum low density lipoprotein cholesterol (LDL-C) levels was not evaluated by most of these studies [5–8, 12, 13]. Finally, allusion to reference groups and comparison between statin users and non-users could have led to the possible “healthy user” bias [16], attributed to behaviors that are linked to medication adherence which may lead to improved health outcomes independently of the real biological effect of the medication [17]. The current study aims to address these methodological challenges evaluating the association between adherence to statins over time and PD risk based on a large-scale cohort of statin users.

## Materials and methods

### Study population

**Fig 1:**
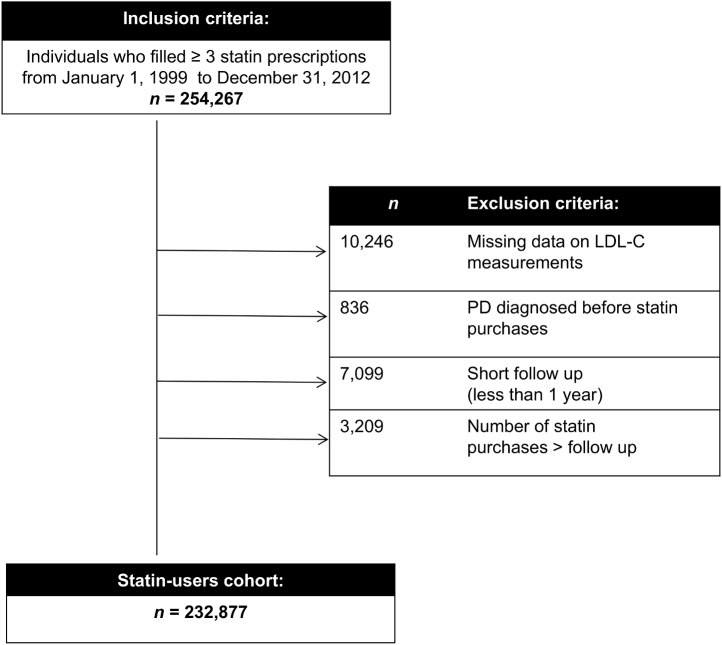
Inclusion and exclusion criteria to establish the statin-users cohort.

A population-based cohort of statin initiators, aged 40-79 at first statin purchase, was derived from the medical database of Maccabi Healthcare Services (MHS), the second largest not-for-profit health maintenance organization in Israel insuring 25% of the population. The cohort initially included 254,267 individuals who filled between January 1^st^, 1999 and December 31^st^, 2012 (study period) at least three new statin prescriptions on seven generic names categorized as cholesterol-lowering drugs according to ATC code C10AA [18]. Of these, 21,390 individuals were excluded due to: missing data on LDL-C levels, PD diagnosis before statin initiation, follow up (FU) shorter than one year, and number of purchases higher than FU years. The final cohort comprised 232,877 individuals with long-term history of data statin adherence, LDL-C levels, gender, and birth date (Fig 1).

### Assessment of changes in statin adherence over time

Statin purchase data was obtained from both MHS and private pharmacies through records of dispensed prescriptions of ATC code C10AA (simvastatin, lovastatin, pravastatin, fluvastatin, atorvastatin, cerivastatin, and rosuvastatin), including dosage, number of pills, and purchasing dates. Statin adherence for each individual was recorded in a chronological order for each 1-year interval (see computation below) from *cohort inception* (time of first statin purchase) to the *end point* (time of PD diagnosis, death, quitting MHS, or end of the study period, whichever occurred first). Statin adherence was lagged by 1 year prior to the end point to avoid protopathic bias (reverse causality). Protopathic bias can occur when PD patients are more likely than controls to start, stop, or change their treatment due to early disease symptoms during the period preceding PD diagnosis [19, 20].

Annual levels of statin adherence were measured by the proportion of days covered (PDC) method, used for measuring medication adherence [21]. We calculated PDC as a continuous variable expressing the total number of months of statin purchases per year divided by the total months of FU during that year (expressed as a 0-100 percentage). Hence, each individual had repeated measures of PDC for their FU period; where the number of the repeated measures was as the number of FU years which varied between individuals. PDC measures were calculated both for all statins and for each separately according to type (lipophilic, hydrophilic) and potency level (low, moderate and high) respective to expected reduction from baseline in LDL-C [22].

### Assessment of LDL-C levels

In Israel, plasma cholesterol profile measurements are routinely screened in men over the age of 35 years and women over the age of 45 years [23]. We assessed all laboratory LDL-C measurements available for cohort members per year concurrent to the years of statin purchases during the study period. Where multiple measurements were available for any given year, the mean annual level of LDL-C was calculated for each chronological FU year. The mean LDL-C value one year before statin purchase was defined *as baseline level*. We followed a single imputation approach [24] to avoid potential selection and measurement biases [24, 25] arising from missing LDL-C values. According to the order of the missing values, we applied one of these methods: regression prediction, last observation carried forward, or first observation carried backward. Hence, each individual had full repeated measures of LDL-C levels for their FU period.

### PD assessment

PD incidence was assessed using an established anti-parkinsonian drug (APD) tracer algorithm [26] and was refined by several selection criteria (we excluded patients with fewer than three consecutive monthly purchases of APD and those treated by bromocriptine, cabergoline or amantadine only). The purchased APD could be any of 17 generic APDs categorized as dopaminergic agents (ATC code N04B) [18] and available in Israel during the study period. Accuracy level of PD assessment was defined as *definitive*, *probable*, or *possible* based on the profile of APD purchases, age at first purchase, purchase density, and length of FU period [26]. Algorithm validation was first accomplished through the comparison of the accuracy level to the gold standard of diagnosis conducted by a neurologist specializing in movement disorders (found highly sensitive at 93%) and subsequently by reviewing PD diagnosis (ICD-9-CM code 332) in medical files and outpatients visits (clinical records). Patients with diagnosis of parkinsonism, gait disorders, essential tremor and non PD-related dyskinesia/spasticity were excluded (3%). All *definitive* PD patients had diagnosis of PD in their clinical records.

### Statistical analysis

We report descriptive statistics including mean, standard deviation (SD), median, and inter-quartile range (IQR) for continuous variables. Due to the nature of repeated measures, changes in annual statin adherence were evaluated using mixed-effects models. The dependent variable was the annual PDC measure (continuous) while the independent variable was the year of FU (categorical).

To evaluate the association between annual statin adherence and the risk of PD, we used Cox proportional hazards models with time-dependent covariates to estimate adjusted Hazard Ratio (HR) with 95%CI. The time scale was the FU years, lagged one year prior to the end point, and the two time-dependent variables were: annual measure of PDC and annual level of LDL-C. The non-time-dependent variables were age at first statin purchase (continuous) and LDL-C at baseline (continuous). We applied three models to refined statin adherence: a) PDC of all statins; b) PDC according to statin type; and c) PDC according to statin potency level. All statistical models were stratified by sex, LDL-C levels at baseline (≤160, >160 mg/dL, a cut point defined as high LDL-C levels by the National Institute of Health [27]), and age categories (5-year intervals) to control for possible interactions between these factors and the effects of long-term statin adherence. To estimate an age-pooled HR, log HRs from the age sub-groups were pooled by a fixed effects model (weighted by the standard error).

All statistical analyses were performed using Statistical Package for the Social Sciences (SPSS) version 22 (SPSS Inc., Chicago, IL) and SAS version 9.4 (SAS Institute, Cary, NC).

#### Sensitivity analyses

For all three models, we performed further analysis. First, considering of potential duration effects, we stratified the analysis according to FU time (≤5 years, >5 years). Second, we examined association of PD with statins in individuals who used simvastatin only (~72% of all purchases). Third, PDC was treated as an ordinal variable (3 and 5 categories). Fourth, the 1-year lag time was replaced by 5 years to allow for a biologically meaningful latency time window where PD occurred before the onset of motor disturbances [28]. Finally, we assumed that all individuals in the cohort were continuous users of statins throughout the study period until their end point. We thus followed single imputation of missing values and adopted regression prediction or last observation carried forward.

In addition, in order to evaluate the possible role of LDL-C levels as an intermediate covariate, we compared HRs of statin exposure in models with and without LDL-C levels as a time-dependent covariate. The correlations between coefficients of PDCs and LDL-C levels were also calculated with both variables.

### Ethics

The Institutional Review Boards (IRB, Helsinki committee) of MHS- Assuta Medical Center (No. 2013052) and of Tel Aviv Medical Center (No. 0281-13-TLV) have both approved the protocol of this study. Personal ID numbers, used to link between different databases, were encrypted prior to delivery to the investigators, to insure anonymity of participants. The study involved no direct interaction with patients, therefore informed consent was not required by the IRBs.

## Results

### General characteristics of the cohort

Table 1 lists the basic characteristics of the 232,877 cohort individuals (49.3% men). Age at first statin purchase was slightly lower among men (56.5 ±9.8 years) compared to women (58.7 ±9.2 years). The FU period from cohort inception to the end point lasted in average 7.5 (±3.4) years for men and 7.7 (±3.4) years for women.

**Table 1:** Characteristics of the statin- users cohort (n = 232,877); by sex.

	Men	Woman
	(n = 114,736)	(n = 118,141)
**Age at first statin purchase (years)**		
Mean (± SD)	56.5 (9.8)	58.7 (9.2)
Median (IQR)^a^	55.5 (48.7-63.7)	57.7 (51.9-65.3)
**Follow up period (years)**		
Mean (± SD)	7.5 (3.4)	7.7 (3.4)
Person years	868,873	916,723
**LDL-C levels (mg/dL) at baseline **		
Mean (± SD)	144.8 (32.9)	152.7 (30.8)
Median (IQR)	145.0 (123.2-165.9)	152.3 (133.4-171.2)
**Proportion of days covered (%) during entire follow up [Median (IQR)]**		
All statins	52.9 (27.6-75.4)	51.0 (26.4-73.7)
Lipophilic	39.5 (15.6-67.8)	38.5 (14.6-66.7)
Hydrophilic	19.1 (6.3-45.5)	16.4 (5.6-38.7)
Low potency ^b^	12.5 (3.5-37.7)	14.4 (4.1-40.6)
Moderate potency ^c^	29.5 (10.9-57.7)	27.5 (9.7-55.6)
High potency ^d^	12.3 (4.1-33.3)	9.1 (3.1-25.0)
**PD cases *n* (%)**	1,355 (1.2)	1,195 (1.0)
**Death cases *n* (%)**	9,599 (8.4)	8,011 (6.8)
**Left MHS**^e^ ***n* (%)**	2,417 (2.1)	2,827 (2.4)

^a^IQR = Interquartile range.

^b^ Low potency statins included daily dose of cerivastatin 0.2 mg; fluvastatin ≤40 mg; lovastatin ≤40 mg or 10 mg twice per day; pravastatin ≤40 mg; simvastatin ≤10 mg.

^c^ Moderate potency statins included daily dose of atorvastatin 10 mg; cerivastatin 0.3 mg or 0.4 mg; fluvastatin 80 mg; rosuvastatin 10 mg; or simvastatin 20 mg or 40 mg.

^d^ High potency statins included daily dose of atorvastatin ≥20 mg; lovastatin 80 mg; pravastatin 80 mg; rosuvastatin ≥ 10 mg; or simvastatin 80 mg.

^e^ MHS = Maccabi Health Services.

### Changes in statin adherence over time

During the study period, cohort individuals made 11,429,603 statin purchases, where the lipophilic type (simvastatin, lovastatin, atorvastatin, fluvastatin, and cerivastatin) was the most widely prescribed for both men and women (73.3% and 74%, respectively). Additionally, most cohort individuals used statins of low to moderate potency (25.2% and 57.1% for men and 31.0%; 55.8% for women, respectively). The median (IQR) duration of statin adherence from cohort inception to last purchase were 6.4 (3.3-9.0) years for men and 6.5 (3.5-9.2) for women.

**Fig 2:**
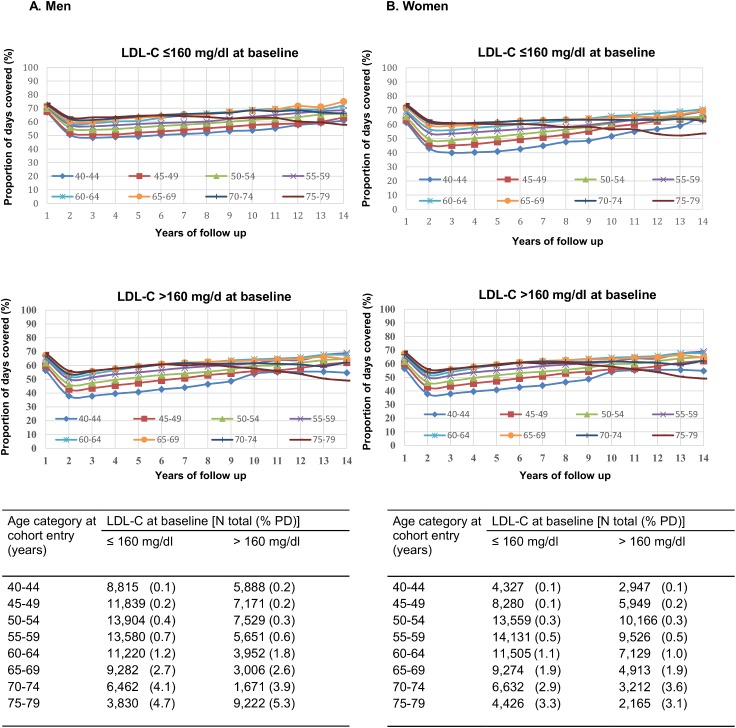
Annual changes in statin adherence* (expressed by proportion of days covered-PDC): according to sex, LDL-C level at baseline and age category (results of mixed models). * First annual PDC measures are based on the FU interval from the first month of statin purchase until the month of December of that calendar year. Last annual PDC measures are based on the FU interval from January of that year until the end point. Other annual PDC measures represent a full calendar year (12 months).

Statin adherence (median PDC for all statins) for the entire study period was slightly higher among men (52.9%) compered to women (51.0%). Some higher adherence range was found for individuals with LDL-C levels ≤160 mg/dL at baseline (PDC 40%- 75%) compared to individuals with LDL-C levels >160 mg/dL (PDC 37%-68%) regardless of sex. Men at all ages with LDL-C levels ≤160 mg/dL at baseline were the most adherent compared to women. In contrast, the youngest study population (40-55 years) of both sexes and regardless of baseline LDL-C level exhibited the lowest statin adherence during the study period. Fig 2 presents annual changes for all statins during FU by sex, LDL-C level at baseline, and age categories.

### Changes in LDL-C levels over time

Median LDL-C levels at baseline were lower among men compared to women (145.0 mg/dL and 152.3 mg/dL, respectively) and over a third of cohort individuals had levels higher than 160 mg/dL (31.2% of men and 38.9% of women). As expected among statins users, LDL-C levels decreased over time, that is, median (IQR) LDL-C levels (mg/dL) at first, third, fifth and seventh years of FU in ascending order were 137.2 (112.2-161.5), 114.2 (91.6-141.2) 110.0 (88.7-136.7) and 105.3 (85.0-131.2) for men and 147.7 (125.2-169.5), 123.1 (100.3-150.0) 119.0 (97.8-146.4) and 116.0 (94.9-141.9) for women.

### PD incidence

Considering at least 1-year of statin adherence during the study period, 2,550 (1.1%) PD cases (1,355 men and 1,195 women) were detected. These reflect an incidence density rate of 1.56 per 1,000 person years for men and 1.30 for women. The average age at first APD treatment was quite similar for both sexes: 72.2 (±8.1) years for men and 72.4 (±7.7) years for women.

PD incident rates were higher among men (1.2%) compared to women (1.0%) and increased significantly with age. For men PD incident rates increased from 0.1% at ages 40-45 to 4.7% at ages 74-79 while for women they increased from 0.1% at ages 40-44 to 3.3% at ages 74-79 (see table in Fig 2).

### Statin adherence over time and PD risk

**Fig 3:**
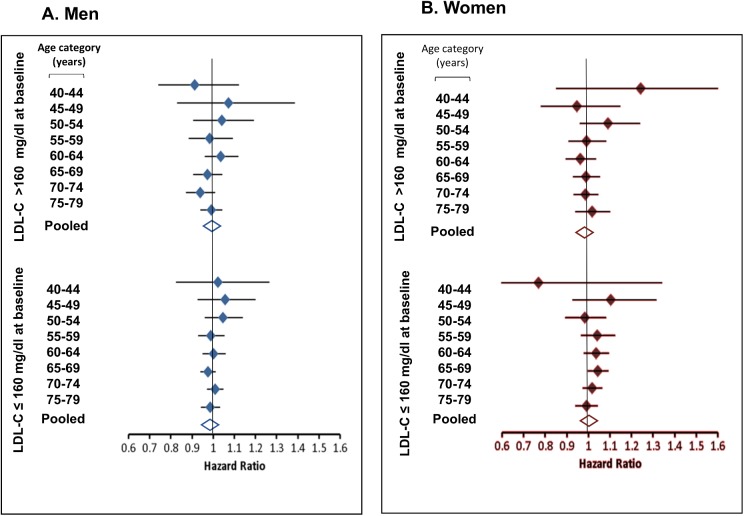
Adjusted Hazard Ratios (HRs) with 95%CIs* for Parkinson’s disease risk associated with annual statin adherence (expressed per 10% increase in proportion of days covered) according to sex, LDL-C level at baseline and age category. *Diamond-shape indicates HR; horizontal lines- 95% CI.

No association was found in the 1-year lagged multivariate Cox regression analysis estimating the HR of annual statin adherence on PD risk given PDCs of all statins. This was true for both men and women and across all age categories (Fig 3). All-risk estimates were close to unity except for the slightly reduced PD risk: HR = 0.77 (95%CI 0.44-1.34) among women aged 40-45 with LDL-C level ≤ 160 mg/dl at baseline. Age-pooled HR (95%CI) for men and women whose LDL-C level at baseline ≤160mg/dL were 0.99 (0.99-1.01), 1.01 (1.00-1.02) while for men and women with LDL-C level >160mg/dL were 0.99 (0.98-1.01), 0.97 (0.98-1.01). Similar null results were obtained when adherence to statins was expressed by type (lipophilic/hydrophilic) or potency level (low, moderate and, high). Sensitivity analyses yielded similar HR estimates for lag period of 5 years, both when PDC was used as a categorical variable and when it was considered with imputation. Analyses considering only the major statin, simvastatin (72% of all purchases) yielded similar null results.

A model employing both time-dependent variables across all age categories yielded correlations between the coefficients (r) at the range of .27–.53. All three Cox models without LDL-C as a time-dependent covariate yielded non-significant effects of statin adherence and PD risk.

## Discussion

Statins are among the most commonly prescribed drugs worldwide [29], rendering it important to determine their potentially effect on PD [30]. Thus, the current population-based study assessed the association between long-term statin adherence and PD risk among statin users. Our null findings suggest no evidence of a neuroprotective effect of statin adherence on PD in this large-scale cohort, considering refined assessments of statin exposure over-time as well as the information on LDL-C levels during the period of statin adherence. Our findings thus largely confirm results from other large-scale epidemiological studies [4–7] as well as a recent meta-analysis study [16]. The latter found no association between statin use and risk of PD in studies that adjusted for cholesterol, suggesting that the apparent protective effect of statins could be explained by statin indication. Some previous observational studies of the statin-PD relationship yielded conflicting results. For example, significantly decreased PD risk was obtained among short-term (1-3 years) statin users [11–13], contrary to estimates based on nigral neuropathological findings or striatal dopamine imaging that suggested a premotor period of at least 5-6 years [19]. Additionally, assessment of statin exposure in most previous studies [4–9, 11–13] were applied for a fixed time interval only, creating a false appearance of drug benefit [31, 32]. The issue of change in statin use was also overlooked despite its suitability in measuring the association between real-life adherence [15,33,34] and the development of neurodegenerative disease which is slowly progressive in its nature [19,28].

Not only do our null findings deny the notion that statin use offers protective effect on PD risk, they render such a possibility highly unlikely. One explanation for this is that while PD occurs mainly among aging populations, the onset of molecular pathologic mechanisms affecting different neuronal tissues throughout the brain and the peripheral autonomic nervous system that are associated with PD may have occurred decades prior to the appearance of cardinal motor signs [35]. Furthermore, *α*-synuclein deposits at the olfactory bulb or brainstem areas that precede the nigral degeneration by 10-30 years [36, 37] suggest that any intervention aiming to slow down or prevent neurodegeneration should occur in the fourth or fifth decade of life rather than the sixth or seventh_._ Another plausible explanation for our null results lies in drug consumption patterns. Where neuroprotective effects of statins were reported, they were obtained mainly from in vitro studies or animal models of PD, under controlled consistent statin use characterized by statin concentrations higher than those used in routine clinical practice [38, 39]. In real life, however, patients may terminate or pause treatment or they may change drugs, which may not be inductive to the replication of reported benefits [22]. Thus, accurate drug adherence measurements, including the consideration of change over time [21, 22], are perquisite for bridging the gap between biological efficacy estimates emerging from experimental trials and the clinical effectiveness recorded in observational studies.

An important strength of the current analysis is the availability of multiple measurements of both statin exposure and LDL-C levels during a period exceeding seven years. This dataset thus allows for the evaluation of the exposure-response statin effect on PD risk. Potential bias related to study outcomes is minimized through the study design, namely, a large-scale population-based follow-up combined with the systematic and comprehensive collection of demographic, laboratory, and pharmacy data at the individual level. Potential information bias is also minimized through the span of exposure history data. Additionally, our use of internal comparisons among individuals who purchased at least three dispensed prescription of statins minimizes healthy user bias. Stratified analysis by LDL-C level at baseline also minimizes indication bias. Both of which may lead to exaggerated potential benefits of statin therapy [16, 17]. Moreover, error variance associated with individual differences is also reduced when allowing individuals to act as their own controls so that the effect of changes in statin adherence on PD outcome is measured within individuals [22].

Study limitations concern statin exposure and the potential inadvertent misclassification of statin use. Firstly, we hypothesized that the dosage of statins taken by individuals in our cohort to be one tablet daily. However, we were unable to ascertain that they were in fact taking the said dosage as they might have been splitting or doubling the quantity, an uncommon practice. Secondly, we evaluated the individual's real drug use based on purchases and as a result we cannot ascertain how the statins were used, if indeed. As well we were unable to point out the reasoning behind the interruption, discontinuation, and restarting of the use of statins in our cohort.

In addition, drug tracer assessment of a PD case could identify non- PD patients due to the difficulty in differentiating Parkinson plus, secondary parkinsonism and some age-related syndromes from PD, solely according to the treatment. However, these cases should be rare and the impact of these limitations is reduced, due to the refined criteria and double validation. Finally, information on factors such as: ethnicity, smoking status, genetic information and comorbidities (e.g. cardiovascular diseases, diabetes mellitus) which might confound the PD associated factors (exposure) was not available in this study.

In conclusion, our large-scale population-based study involving high-quality registry data on refined statin adherence does not support the hypothesis that long-term statin adherence confers protection against PD. Our study exemplified the importance of a refined assessment of statin adherence patterns and the applications of time-dependent modeling techniques to assess whether statins actually affect PD risk. Additional large-scale observational studies employing long-term follow-up periods are needed to further elucidate this point.
